# Film-Thickness Identification Method and Lubrication Characteristic Experiment of Full-Size Water-Lubricated Stern Bearing under Offset Load

**DOI:** 10.3390/s22103670

**Published:** 2022-05-11

**Authors:** Jinjun Li, Wu Ouyang, Qilin Liu, Zhuo Zhang, Yan Zhang

**Affiliations:** 1 School of Transportation and Logistics Engineering, Wuhan University of Technology, Wuhan 430063, China; ljjwhut@whut.edu.cn (J.L.); 250067@whut.edu.cn (Q.L.); zzhuo@whut.edu.cn (Z.Z.); yanzhang@whut.edu.cn (Y.Z.); 2 Reliability Engineering Institute, National Engineering Research Center for Water Transport Safety, Wuhan 430063, China

**Keywords:** water-lubricated stern bearing, full-circumference film-thickness identification, axial orbit, distributed characteristics, lubrication characteristics

## Abstract

Water-lubricated stern bearing (WSB) is a vital part of the ship propulsion-shaft system, and it is of great significance to monitor and analyze its lubrication status through film thickness data to improve the equipment operational reliability. In this paper, a full-size, large length-to-diameter ratio WSB experiment is carried out, and multi-sectional journal displacement data are collected under offset load. Accordingly, a bearing film-thickness identification model is established, which can identify the dynamic film thickness data in the circumferential direction of bearing section by limited measurement points. On this basis, the film thickness distribution of the full bearing is obtained by combining finite element (FE) simulation and particle swarm optimization (PSO) algorithm. The effect of different speeds on the distributed lubrication characteristics of WSB under offset load was systematically analyzed based on film thickness data. Results show that the maximum identification error of the bearing film-thickness identification model is less than 7%. The bearing lubrication state changes dynamically as the speed increases, and the hydrodynamic lubrication effect in the middle of the bearing is enhanced. The area of each lubrication sub-region varies nonlinearly. Research results are instructive for further determine the service life of the shaft system.

## 1. Introduction

The propulsion-shaft system is an important part of a ship’s power system, and the stern bearing is an essential equipment of this system [1]. The stern bearing is used to support the stern shaft and propeller to ensure the regular operation of the propulsion-shaft system. Most ship stern bearings are water lubricated, and seawater is adopted as the medium for lubrication and cooling, thereby improving the system’s reliability. It has become a resource-saving, environmentally-friendly, and low-noise lubrication solution and is extensively used in civilian ships [2]. However, the low load-carrying capacity of water and the nonlinear deformation characteristics of the bearing lining lead to a very complex lubrication state of ship WSB in operation [3].

Film thickness is an important parameter for characterizing the lubrication state of WSB. Due to the low-speed and heavy-load operating conditions of water-lubricated bearings and the low viscosity characteristics of water, the bearing film thickness is often too thin and causes contact between the shaft and bearing surface, leading to excessive wear and friction during long-term operation [4,5]. In recent years, scholars have paid attention to the effect of film thickness state on the lubrication performance of water-lubricated bearings. Rao [6] derived the film-thickness and pressure equations for a journal bearing at steady-state according to the control equations with partial slip on the bearing surface. The related performance of concentric radial plain bearings has already been analyzed, but a particular gap exists under the actual operating condition of the bearing. Chasalevris et al. [7,8] used the analytical method of Reynolds equation for finite-length sliding bearings to obtain the analytical solution of liquid-film impedance force. They analyzed the main parameters of sliding bearing, such as eccentricity, minimum film-thickness position, stiffness, and damping coefficient, which were valuable for analytically solving the lubrication problem of finite journal bearings. Jin et al. [9] investigated the effect of each design parameter on the nonlinear dynamic performance of water-lubricated bearings considering static and non-static loads and obtained the optimized parameters that maximized the minimum film thickness. Liu et al. [10] considered the effect of elastic deformation on the lubrication state of water-lubricated bearings and analyzed the variation of parameters such as maximum pressure value and minimum oil film thickness under different bearing clearance structures. Lv et al. [11,12] indicated that large bearings’ maximum oil film thickness was much greater than the minimum nominal oil film thickness, and local turbulence might occur. Results of calculations by the finite-difference method also showed that turbulence significantly affected the bearing friction coefficient and the minimum oil film thickness.

Film-thickness prediction and measurement are the two main contents for analyzing bearing-lubrication characteristics. Film-thickness prediction techniques are suitable for many bearing-design applications. However, more direct measurement methods are often required for high-precision or high-load systems [13]. Many film-thickness measurement methods are available, such as electrical methods [14,15,16], optical methods [17,18,19,20], and ultrasonic methods [21,22,23]. Among them, the eddy-current method [24,25] is one of the most applied methods in industrial settings given its good working reliability, high sensitivity, and strong anti-interference ability. Scholars have tried to explore the information of film thickness status and lubrication status of bearings during operation through experiments. Sun [26] selected two sections in the axial direction of the journal bearing and arranged two eddy-current sensors in each section to analyze the effects of load and length-to-diameter ratio on bearing parameters such as film thickness. Nevertheless, the number of measurement points is insufficient, and the information on the film-thickness distribution along the axial direction is unknown. Chatterton et al. [27] conducted an experimental study on the performance of journal bearings under severe operating conditions. They selected several experimental conditions under different static loads and rotational speeds to demonstrate the correlation law between film thickness and other status parameters. Xie et al. [28,29] arranged four displacement sensors uniformly along the bearing circumference to monitor the bearing film thickness and bi-directional vibration. And explained the transformation mechanism of the lubrication state of the water-lubricated bearing. Wodtke et al. [30] studied the thermal phenomena during the operation of WSB by theory and experiment and pointed out that thermal deformation favors film thickness generation when the bearing casing is considered for heat transfer. Overall, most reported bearing experimental conditions are ideal, and the test bearing size is less than one-third of the actual ship bearings, which is not enough to simulate actual operating conditions. Therefore, the correspondence of lubrication characteristics between full-size bearings and reduced ratio bearings remains to be revealed.

Most current researchers arrange limited measurement points for bearing film-thickness identification. However, owing to the deformation, wear, and other factors, the film-thickness states at various locations within the bearing are not consistent during actual operation. Real-time monitoring and analysis of bearing film-thickness distribution are difficult to achieve through limited measurement points. Based on the above status, a lubrication characteristic experiment of full-size WSB is carried out in this work. The actual operating conditions of the ship’s shaft system are simulated and the journal displacement data were collected to provide an experimental basis for conducting research on the dynamics of water-lubricated bearings with offset load and large length-to-diameter ratio. In addition, a bearing film thickness identification method is proposed, which can identify the dynamic film thickness data of bearing circumferential direction by limited measurement points. The bearing lubrication characteristics at each speed were studied based on the film thickness data, which is of great value in determining the WSB service status.

## 2. Experimental Apparatus

This subsection constructs a WSB test rig with a full-size and large length-to-diameter ratio bearing. The actual operating conditions of the ship’s stern bearing are simulated.

### 2.1. Test Rig and Test Bearings

**Figure 1:**
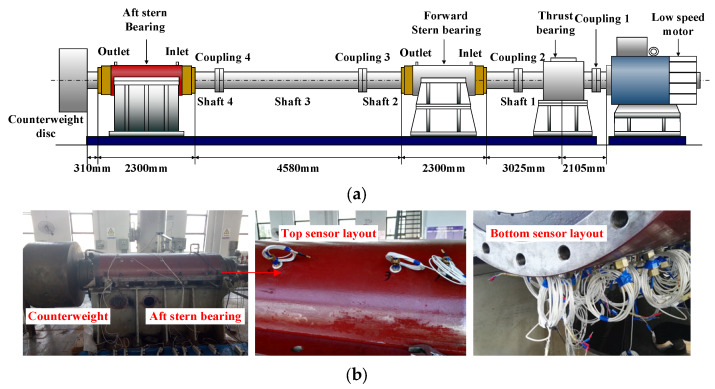
Test rig: (**a**) schematic of the structure and (**b**) test bearing.

The test rig used in this paper is shown in Figure 1. It comprises a low-speed motor, a thrust bearing, a front bearing, a stern bearing, a counterweight plate, and three rotating shafts. Among them, the thrust bearing is an oil-lubricated bi-directional thrust bearing. The front and stern bearings are water-lubricated, and the stern bearing is the main test object of this paper. The test is primarily conducted at the end of the test rig, which includes the stern shaft, stern bearing, and mechanical seal components, whereas the stern shaft is connected to the intermediate shaft by using a flange. The test rig simulates a natural ship-propulsion system with a shaft diameter of over 300 mm, a speed range of 0~300 r/min, and a counterweight plant mass of 7.5 t.

A bearing water lubrication system is set up next to the test rig, which is connected to the water-lubricated bearings. The upper right end of the bearing is connected to the water inlet and the upper left end of the bearing is connected to the water outlet. The inlet and outlet pipes of the lubrication system are equipped with pressure gauges, flow gauges and temperature sensors, which allow the water supply pressure and flow rate to be adjusted as needed.

**Figure 2:**
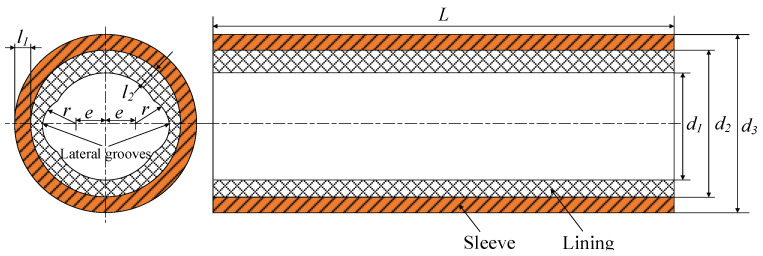
Structural diagram of the test bearing.

The stern bearing, including the lining and the sleeve, is the test bearing. The bearing structure is shown in Figure 2. The sleeve material is stainless steel, and the lining is a polymer composite material with a modulus of elasticity of 2320 MPa, Poisson’s ratio of 0.327, and density of 1300 kg·m^–3^. Both sides of the lining have a lateral groove with radius *r*, and the distance between the centers of the lateral groove and the bearing is *e*. The bearing length-to-diameter ratio is 3.7, and the shaft diameter is 323 mm. In the figure, *l*_1_ is the lining thickness, and *l*_2_ is the sleeve thickness. The main structural parameters are shown in Table 1.

### 2.2. Sensor Settings

**Figure 3:**
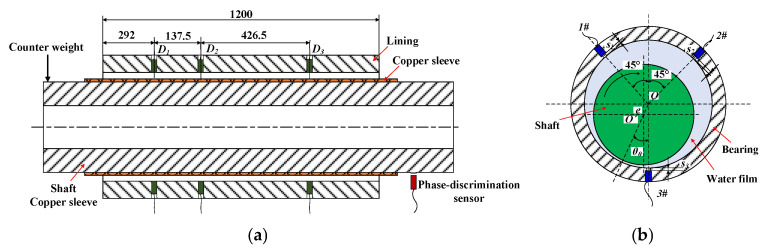
Eddy-current sensor arrangement: (**a**) axial arrangement scheme; (**b**) circumferential arrangement scheme.

Film thickness is the object of study; it means the clearance distance between the rotating shaft and the sleeve in the water-film carrying area [31]. The film thickness is measured by indirect method, multiple eddy-current sensors are arranged on the bearings to obtain the distance between the sensor probe and the rotating shaft at the measurement point. Specific geometric equations and test procedures deduce the full circumferential film thickness. The sensor arrangement is shown in Figure 3. Three test sections (D_1_~D_3_) are set up on the bearing, and three through holes are machined in each section and eddy-current sensors are installed. The arrangement scheme is the same for each section; 1# and 2# sensors are symmetrically distributed at the top of the bearing with an angle of 45° to the vertical direction, and 3# sensor is arranged at the lowest point of the bearing vertical direction. The arrangement scheme of the remaining sections is the same as that of D_1_. In the figure, O is the bearing center, O′ is the rotating shaft center, *θ*_0_ is the eccentricity angle, e is the eccentricity distance, and *S*_1_, *S*_2_, and *S*_3_ are the distances between the three sensor probes and the bearing inner surface.

**Figure 4:**
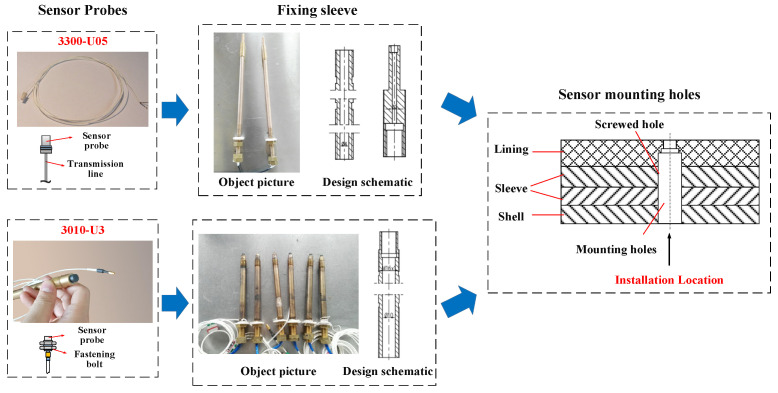
The eddy-current sensors used in the test.

The sensors used in the test are shown in Figure 4, and the sensor parameters are shown in Table 2. #1 and #2 sensors are eddyNCDT 3010-U3 high-precision eddy-current sensors (Micro-Epsilon America, Raleigh, NC, USA) with 3 mm measuring range and 0.15 μm resolution. The sensor probe has connection threads and is screwed into the fixing sleeve and then mounted in the bearing hole. #3 sensors are eddyNCDT 3300- EU05 pressure-resistant high-precision eddy-current sensors with 0.5 mm measuring range and 0.025 μm resolution, which can meet accuracy and pressure-resistance requirements. The sensor has no connection structure, so a specialized fixing sleeve is designed and encapsulated with epoxy resin. The position of the eddy-current sensor needs to be adjusted during installation so that the distance between the probe and the shaft surface is near the median of the sensor range to ensure that the measurement accuracy meets the requirements.

The signal-test system comprises a data-sensing module, a data-acquisition module, and a signal-processing module. Among them, the data-sensing module includes sensors, conditioners, calibrators, and power-supply meters. The data-acquisition module uses the OR38 test analyzer from OROS, which supports up to 32 signal-input channels, allowing simultaneous analysis of multiple channels of input information in different modes with stable operation and a high signal-to-noise ratio.

## 3. Bearing Film-Thickness Identification Model

**Figure 5:**
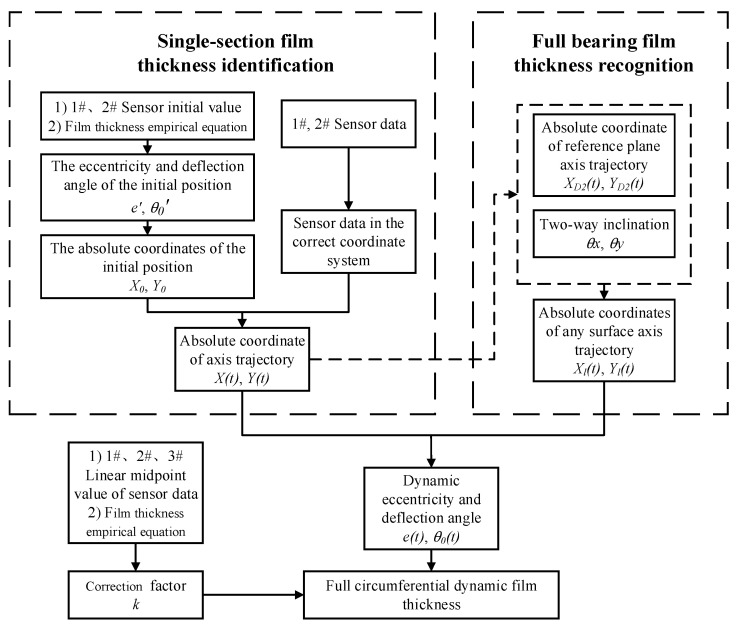
Flow chart of the full-circumference film-thickness identification method.

The flow chart of the film-thickness identification method is shown in Figure 5. This method identifies the dynamic film thickness at each point of the bearing circumference by calculating the relative position of the shaft center to the bearing.

The initial position of the shaft center measured at the time of stable bearing operation is selected as the reference point. According to the empirical equation of film thickness, the following relationship exists between sensor data and film thickness at the initial moment.

(1) $$\left\{ \begin{array}{l} a_{1}{}^{\prime }-S_{1}=c+e^{\prime }*\cos(315{}^{\circ}-\theta_{0}{}^{\prime })\\ a_{2}{}^{\prime }-S_{2}=c+e^{\prime }*\cos(45{}^{\circ}-\theta_{0}{}^{\prime }) \end{array}\right.\;$$

where $a_{1}{}^{\prime }$ and $a_{2}{}^{\prime }$ are the test values of sensors #1 and #2 at the reference point, *S*_1_ and *S*_2_ are the distance between the two sensor probes and the bearing inner surface, *c* is the radius clearance, $e^{\prime }$ is the eccentricity distance of the reference point, and $\theta_{0}{}^{\prime }$ is the eccentricity angle of the reference point. The equation is solved to obtain the absolute coordinate values $X_{0}$ and $Y_{0}$ of the reference point in a right-angle coordinate system.

(2) $$\left\{ \begin{array}{l} X_{0}=\frac{c+S_{2}-a_{2}{}^{\prime }}{\sqrt{1+\left(\frac{a_{1}{}^{\prime }-S_{1}-c}{a_{2}{}^{\prime }-S_{2}-c}\right)}}*\sin\left[45{}^{\circ}-\arctan\left(\frac{a_{1}{}^{\prime }-S_{1}-c}{a_{2}{}^{\prime }-S_{2}-c}\right)\right]\\ Y_{0}=\frac{c+S_{2}-a_{2}{}^{\prime }}{\sqrt{1+\left(\frac{a_{1}{}^{\prime }-S_{1}-c}{a_{2}{}^{\prime }-S_{2}-c}\right)}}*\cos\left[45{}^{\circ}-\arctan\left(\frac{a_{1}{}^{\prime }-S_{1}-c}{a_{2}{}^{\prime }-S_{2}-c}\right)\right] \end{array}\right.\;$$

The absolute coordinates of the axial orbit are derived from the following equation.

(3) $$\left\{ \begin{array}{l} X(t)=X_{0}+\left[a_{2}{}^{\prime }-a_{2}\left(t\right)\right]\\ Y(t)=Y_{0}+\left[a_{1}{}^{\prime }-a_{1}\left(t\right)\right] \end{array}\right.$$

where $a_{1}{}^{\prime }\left(t\right)$ and $a_{2}{}^{\prime }\left(t\right)$ are the measurement data of sensors #1 and #2 at any moment. The dynamic film thickness is calculated from Equation (4).

(4) $$h\left(\theta ,t\right)=c+\sqrt{X{\left(t\right)}^{2}+Y{\left(t\right)}^{2}}*\cos\left[\theta -\arctan\frac{Y\left(t\right)}{X\left(t\right)}\right]$$

where θ is the angle of the position to be solved; and h(θ,t) is the film thickness at any moment and position.

In actual operation, owing to wear, deformation, and other conditions, the empirical formula is often difficult to accurately reflect the actual film-thickness state. Thus, a correction factor *k* is introduced to optimize the identification method, and the following equation is established.

(5) $$\left\{ \begin{array}{l} \bar{a_{1}}-S_{1}=c+\bar{e}*\cos\left(315{}^{\circ}-\bar{\theta_{0}}\right)+k\\ \bar{a_{2}}-S_{2}=c+\bar{e}*\cos\left(45{}^{\circ}-\bar{\theta_{0}}\right)+k\\ \bar{a_{3}}-S_{3}=c+\bar{e}*\cos\left(180{}^{\circ}-\bar{\theta_{0}}\right)+k \end{array}\right.\;$$

where $\bar{a_{1}}$, $\bar{a_{2}}$, and $\bar{a_{3}}$ are the linear midpoint values of the periodic data obtained from three sensors of the same section measured. Under the conditions of stable bearing operation, the condition parameters of the bearing are stable during the experimental-measurement period. The correction factor *k* is obtained by solving the above equation.

(6) $$\left\{ \begin{array}{l} \bar{\theta_{0}}=\arctan\frac{1}{1-2\left(\frac{\bar{a_{1}}-S_{1}-\bar{a_{3}}+S_{4}}{\bar{a_{1}}-S_{1}-\bar{a_{2}}+S_{2}}\right)}\\ \bar{e}=\frac{\bar{a_{1}}-S_{1}-\bar{a_{2}}+S_{2}}{-\sqrt{2}\sin\bar{\theta_{0}}} \end{array}\right.\;$$

(7) $$k=\bar{a_{1}}-S_{1}-\bar{e}*\cos\left(45{}^{\circ}+\bar{\theta_{0}}\right)-c$$

**Figure 6:**
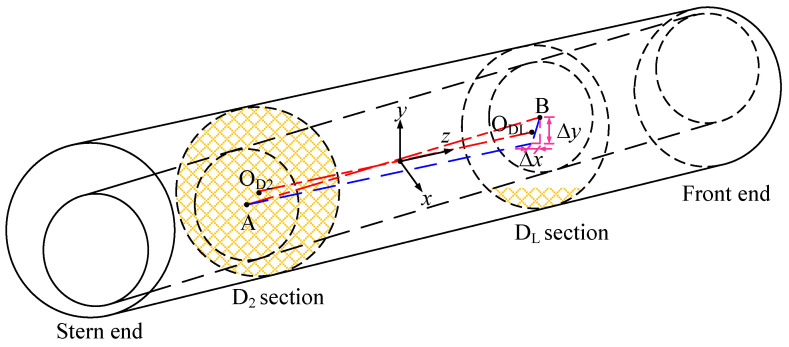
Three-dimensional schematic of bi-directional bearing tilt.

The above derivation can obtain the dynamic film thickness data in the circumferential direction of the bearing section. The film thickness of the non-test section can be deduced by the following method, provided that the film thickness and axial-diameter position of the test section have been obtained. As shown in Figure 6, the relative position relationship between the sections can be expressed by the axial distance and bi-directional tilt angle. Section D_2_, located in the middle of the bearing, is selected as the reference surface, and O_D2_ (bearing midpoint of the section ) is the axial origin. The rotation angle of the journal around the horizontal *x*-axis at this point is *θ_x_*, which is the journal vertical inclination. The rotation angle around the vertical *y*-axis is *θ_y_*, which is the horizontal journal inclination. A and B are the shaft centers of the two sections. The dynamic coordinates of the axial orbit of any section can be obtained according to the spatial state of the axial diameter.

(8) $$\left\{ \begin{array}{l} X_{l}\left(t\right)=X_{D2}\left(t\right)+\Delta l*\tan\theta_{x}\left(t\right)\\ Y_{l}\left(t\right)=Y_{D2}\left(t\right)+\Delta l*\tan\theta_{y}\left(t\right) \end{array}\right.$$

where $X_{D2}\left(t\right)$ and $Y_{D2}\left(t\right)$ are the axial orbit coordinates of section D_2_, and Δl is the difference between the axial positions of section D_2_ and section D_L_.

The dynamic film thickness of the full bearing is deduced by combining the single-section film-thickness identification method.

(9) $$h\left(\theta ,l,t\right)=c+\sqrt{X_{l}{\left(t\right)}^{2}+Y_{l}{\left(t\right)}^{2}}*\cos\left(\theta -\arctan\frac{X_{l}\left(t\right)}{Y_{l}\left(t\right)}\right)+k$$

## 4. Experimental Procedure

To study the lubrication conditions of stern bearing journals under different operating conditions, multi-sectional film-thickness measurement of a full-size stern bearing was performed. The test conditions are shown in Table 3.

Before the test, a counterweight plate was installed at the end of the cantilever, the height of the front-end bearing was adjusted to 0, water was injected into the bearing gap until the water supply pressure reached 0.13 MPa, and a 24 h water-up test and a 12 h break-in test were conducted. After the break-in test, the measured bearing radius clearance was approximately 0.55 mm. During the test, the test speed was reduced from 215 rpm to 15 rpm, and each 10 rpm decrease was used as a test condition lasting for 1 h. Film-thickness data were measured and recorded using the signal-test system and eddy-current sensors.

## 5. Results and Discussion

This section derived bearing film-thickness data using the film-thickness identification model. The film-thickness distribution and lubrication state law of WSB under various operating conditions are analyzed in depth.

### 5.1. Test Section Film-Thickness Analysis

**Figure 7:**
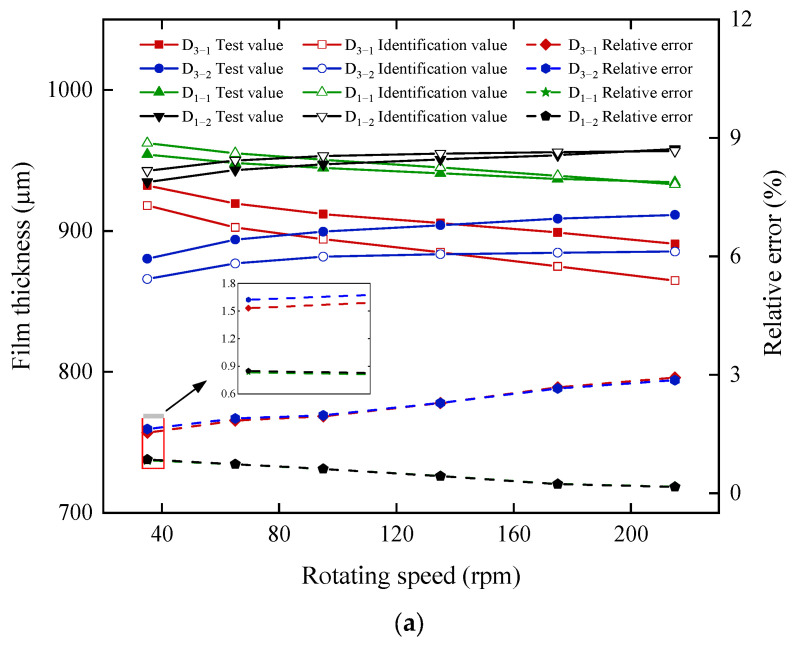

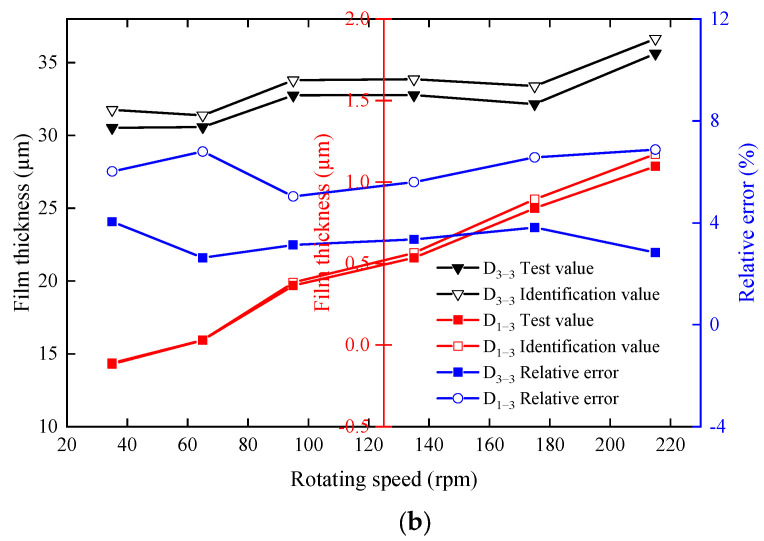
Comparison of test and identification film-thickness results: (**a**) Data of #1 and #2 sensors in each section and (**b**) data of #3 sensor in each section.

The identification method proposed in this study enables the identification of bearing circumferential film thickness using data from measurement points. Figure 7 compares the thickness of the test and identification film for six measurement points in two selected test sections. The data of sensors #1 and #2 in two test sections are shown in Figure 7a, the maximum relative errors corresponding with the four measurement points are 2.93%, 2.86%, 0.83%, and 0.85%, respectively. With the increase of speed, the relative error of section D_1_ decreases and the relative error of section D_3_ increases. The data of sensors #3 in two test sections are shown in Figure 7b, the maximum relative errors corresponding with the two measurement points are 6.87% and 4.04%. The above results show that the identification results well match the test results, and the trend of test-film thickness and identification-film thickness remains the same.

**Figure 8:**
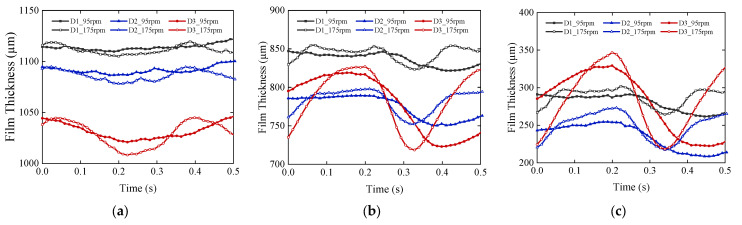

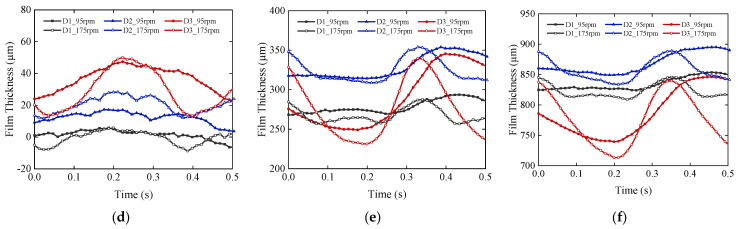
Film thickness at each angle of sections D_1_~D_3_ at 95 rpm and 175 rpm: (**a**) 0°; (**b**) 60°; (**c**) 120°; (**d**) 180°; (**e**) 240°; and (**f**) 300°.

The sensors are arranged at 45°, 180°, and 315°, respectively, in the test sections; the highest point in the vertical direction is defined as 0°, the angle increases in the clockwise direction, and the angles described later are defined according to this rule. The dynamic film-thickness values at non-test points are obtained using the film-thickness identification method. Figure 8 shows the dynamic film-thickness data for multiple angular positions in each section for two selected operating conditions of 95 and 175 rpm. Film thickness fluctuations amplitude increase with the increase of speed. The film-thickness fluctuations on both sides of the bearing are larger than that on the middle. The film-thickness curve of section D_1_ is irregular in shape and has spikes, whereas that of section D_3_ is closer to the sinusoidal shape, indicating that section D_1_ is in friction and section D_3_ is in better lubrication condition.

The film thickness at the lower end of D_1_ section has negative value. The reason is that D1 section is close to the ballast end of the bearing, its lubrication condition is very poor, and there is large elastic deformation of the liner, resulting in rough contact between the journal and the lining in this section. This also results in the distance a_3_ between the #3 sensor and the journal being less than the #3 sensor mounting distance S_3_ and the test film thickness and identification film thickness being negative. This negative value reflects the degree of elastic deformation and wear of the lining.

**Figure 9:**
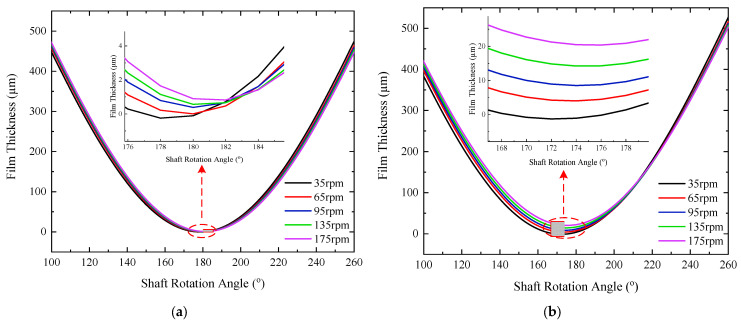

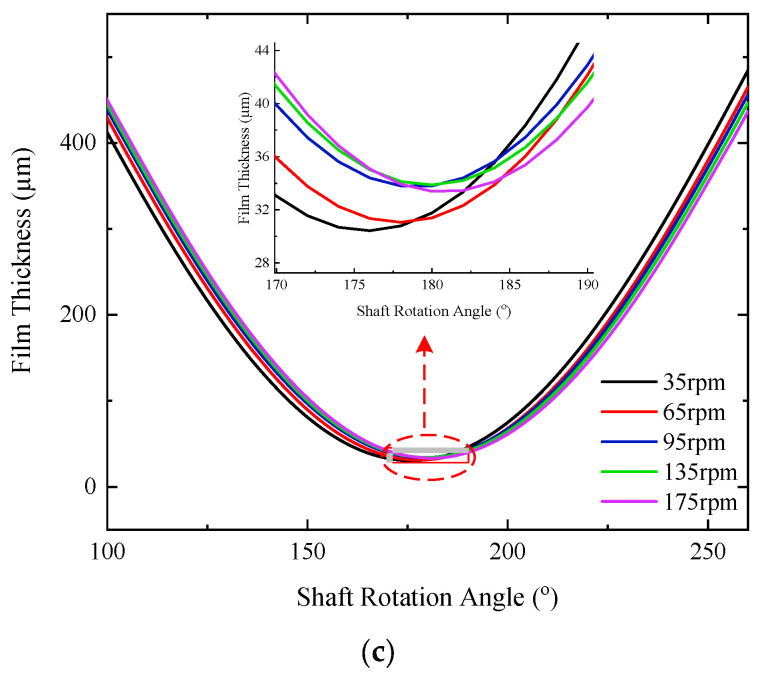
Film thickness-circumference distribution of test sections at different rotational speeds: (**a**) D_1_ section; (**b**) D_2_ section; and (**c**) D_3_ section.

**Figure 10:**
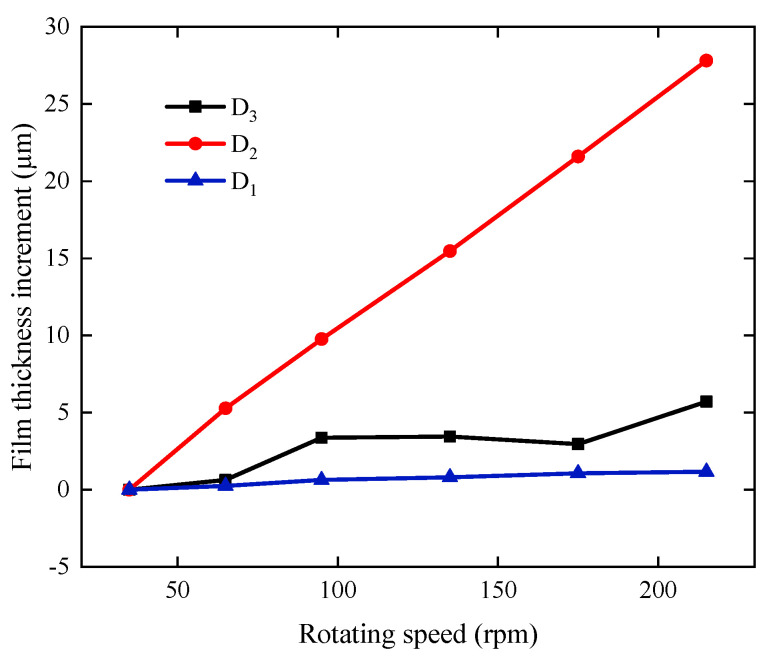
Minimum film-thickness increases for each section based on 35 rpm.

Figure 9 compares the circumferential distribution of film thickness of the test sections within the speed range of 35 rpm to 175 rpm. Figure 10 shows the minimum film thickness increases for each section based on 35 rpm. Under the effect of offset load, each bearing section shows different film-thickness distribution patterns. As the speed increased from 35 rpm to 215 rpm, the minimum film thickness of the three sections increases by 1.17, 27.82, and 5.71 µm, respectively. Section D_1_ is close to the ballast end, which is subject to a large suppression effect, the variation in film thickness is much smaller than the other two sections. The minimum film thickness of section D_3_ barely increases at speeds greater than 95 rpm. The reason may be that the ballast action causes vertical deflection of the journal, raising the journal at D_3_ section and forming hydrodynamic lubrication at lower speeds, and the film thickness does not change significantly with increased speed.

### 5.2. Journal Position Status Analysis

**Figure 11:**
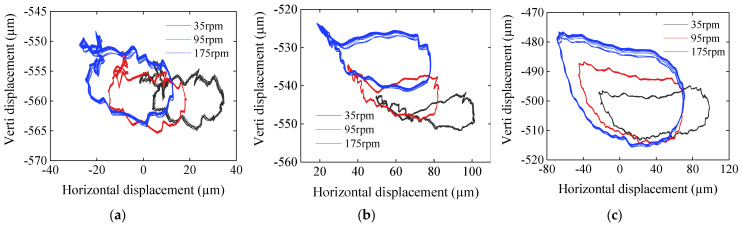
Comparison of axial orbits at different speeds: (**a**) D_1_ section; (**b**) D_2_ section; and (**c**) D_3_ section.

To further explore the intrinsic factors of bearing water-film distribution law, the journal position status is analyzed. The variation in the axial orbit of the three tested sections with respect to the rotational speed is shown in Figure 11. The shaft vibration of section D_1_ is slight, resulting in small changes in the size and shape of the shaft orbit, whereas the shape of the orbit presents “8”. Thus, the bearing is in boundary lubrication or mixed lubrication state. Compared with section D_1_, with increased speed, the orbit shape of section D_2_ is gradually smooth, and the lubrication state gradually changes to hydrodynamic lubrication. The orbit area of section D_3_ significantly increases with the increase of speed, and the orbit is smooth at all speeds, indicating that it is in the hydrodynamic lubrication state.

**Figure 12:**
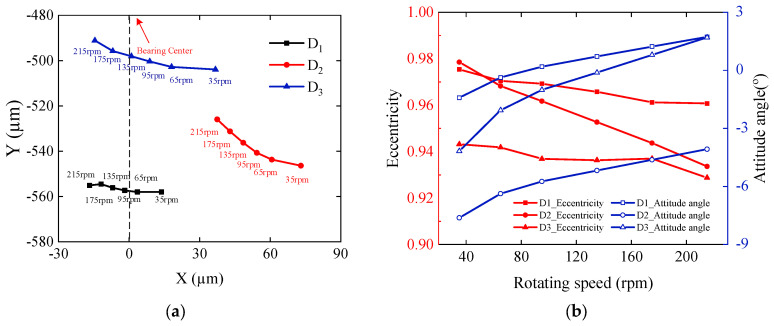
Journal position status at different speeds: (**a**) Axis position and (**b**) Eccentricity and eccentricity angle.

The journal position status of the test sections at different rotational speeds are shown in Figure 12. With increased rotational speed, each section moves to the upper left, eccentricity decreases, and eccentricity angle increases. The eccentricities of the three sections decrease by 1.45%, 3.56%, and 0.66%, respectively, with the speed increasing from 35 rpm to 175 rpm. The axis heights increase by 0.525%, 3.741%, and 2.540%, respectively, indicating that the increase in speed intensifies the deflection of the journal. Comparing the axial-position change pattern of three sections, it can be seen that the change amplitude of the axial orbit in the horizontal direction is larger than that in the vertical direction. The relative differences between the three sections’ horizontal and vertical displacement are 81.33%, 49.69%, and 86.44%, respectively, with the speed increasing from 35 rpm to 175 rpm. From the above analysis, it is clear that the journal in the stern bearing bore presents a complex bending state. The stern bearing has multiple lubrication states, each lubrication region along with the axial distribution.

### 5.3. Bearing Three-Dimensional Film-Thickness Analysis

To further study the bearing distributed lubrication characteristics, it is necessary to identify the film thickness of the whole bearing. The bearing lubrication status can be judged according to the following method:

(10) $$\lambda =\frac{h_{\min}}{\sqrt{R_{q1}^{2}+R_{q2}^{2}}}=\frac{h_{\min}}{1.25\sqrt{\delta_{1}^{2}+\delta_{2}^{2}}}\;$$

where *h_min_* is the bearing minimum film thickness, *R_q_* is the root-mean-square deviation of the contact surface profile (the root-mean-square deviation *R_q_* has the following approximate relationship with the arithmetic mean deviation *δ*: *R_q_* = 1.20*δ*~1.25*δ*), and *λ* is the film-thickness ratio, which is an empirical factor that considers the combination of shape error and mounting error in bearing manufacturing.

(1) When *λ* > 3, the bearing is in a hydrodynamic lubrication state, which can avoid abrasion and wear;
(2) When 1 < *λ* < 3, the bearing is in a mixed lubrication state, and the probability of abrasion on the bearing working surface is high;
(3) When *λ* < 1, the bearing interface is in the boundary lubrication state, and abrasion and wear are bound to occur.

According to the general processing of a polymer-composite stern bearing, the bearing roughness *δ*_1_ in this experiment is 1.6 μm, and the surface roughness *δ*_2_ of the shaft copper sleeve is 0.8 μm. Substitute into Equation (10), when *λ* = 1, then *h_min_* = 2.24 μm; when *λ* = 3, then *h_min_* = 6.71 μm.

**Figure 13:**
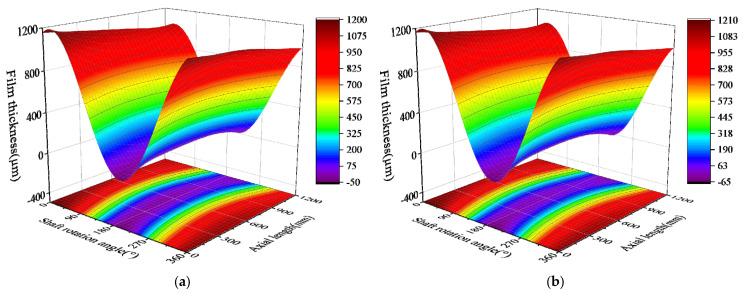

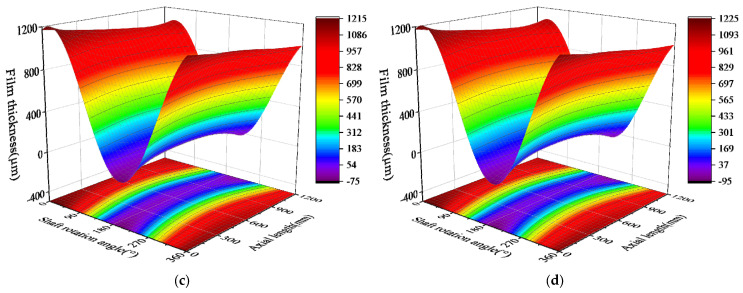
Three-dimensional film-thickness distribution at different speeds: (**a**) 35 rpm; (**b**) 65 rpm; (**c**) 95 rpm; and (**d**) 135 rpm.

The minimum film thickness of each section is determined by calculating the three-dimensional film thickness data of the bearing. According to the film thickness identification method proposed in Section 3, to obtain the full-bearing film-thickness data, it is necessary to calculate the axis position data for each section. The axis position of the test sections can be accurately obtained, and the axis position of the non-test sections can be deduced by combining the shaft-bending curve. The shaft-bending curve was obtained by fitting the axial position data of the three test sections using the least-square method, and the film thickness data of the full bearing was deduced by combining the film thickness identification method. Figure 13 shows the three-dimensional film-thickness distribution at different speeds. From the film-thickness projection, it can be seen that the film-thickness contour is not parallel to the axis. The axis is clearly bent in the horizontal and vertical directions. However, there are negative values of film thickness at the beginning and end of the bearing, the reason may be that there are few test points, which makes it difficult to correctly reflect the real state of the shaft bending curve in the test-data fitting results.

The reason for the negative values of the above 3D film thickness is the large error in the fitted shaft-ending curve. To improve the accuracy of the identification results, the shaft-bending curve needs to be optimized. The following steps are included. FE simulation is used to obtain the theoretical shaft-bending curve function under the corresponding working conditions. The three measured sections data is brought into the PSO algorithm, and the difference between the root-mean-square error and the correlation coefficient is used as the objective function to obtain the coefficient values of the theoretical shaft-bending curve function. The three-dimensional film thickness of the bearing is deduced from this optimized shaft-bending curve.

The simulation method includes the following steps. A three-dimensional model of the test bearing is established, and other bearings are simulated by spring. The model is meshed using the sweep method, and the grid unit is selected as the solid unit. The contact mode between shafts is set as bonding. The bearing support mode is fixed supporting. The stiffness of the spring simulated bearing is 10^9^ N/m. Gravitational acceleration is applied to the model, and the concentrated mass is used to simulate propeller loads. The weight of the concentrated mass is 7.5 t.

**Figure 14:**
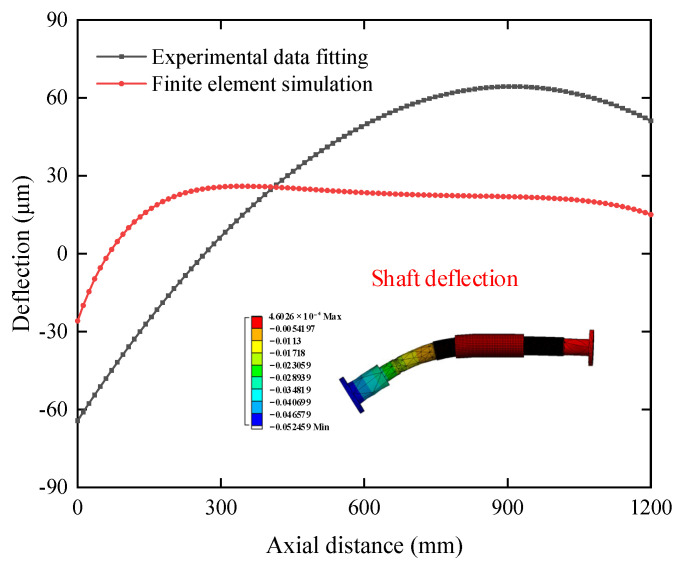
Comparison of shaft-bending curves from FE simulation and least square fitting.

Figure 14 compares the theoretical shaft bending curve obtained by FE simulation with the shaft bending curve obtained by least-square fitting of experimental data. The maximum deflection of the stern both occurs at the ballast end, but the curve of FE simulation is less curved and more consistent with the actual operating conditions.

**Figure 15:**
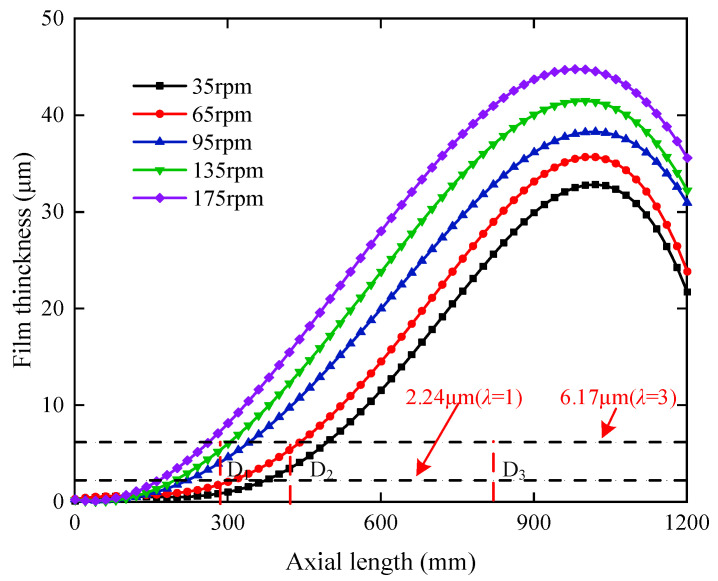
Minimum film-thickness distribution along the axial direction at each speed.

Based on the optimized shaft-bending curve, the film thickness of the full bearing is calculated, and the minimum film-thickness distribution along the axial direction at each speed is shown in Figure 15. The three test sections are located at 292 mm, 429.5 mm, and 856 mm, respectively, from the ballast end of the bearing. The minimum film thickness of section D_1_ is greater than 6.17 μm with increased speed to 175 rpm, indicating a change from mixed lubrication state to hydrodynamic lubrication. Section D_2_ changed to hydrodynamic lubrication after the speed increased to 95 rpm. Section D_3_ is in hydrodynamic lubrication at five measured speeds. The above results verify the analysis of the bearing-lubrication status in Figure 9 and Figure 10. As the shaft is deflected by ballast, the ballast section of the bearing is in boundary lubrication and mixed lubrication, and the rest is in hydrodynamic lubrication. Table 4 shows the range of the three lubrication sub-regions at each speed. It can be seen that during the increase of speed from 35 rpm to 175 rpm, the boundary lubrication sub-region is reduced by 56.27%, the mixed lubrication sub-region is reduced by 18.33%, and the hydrodynamic lubrication sub-lubrication area is expanded by 33.05%. Each lubrication sub-region area varies nonlinearly.

## 6. Conclusions

In this work, a lubrication characteristic experiment of WSB is carried out. A full-size bearing with large length-to-diameter ratio is used as the test bearing, and the actual operating conditions of the ship’s stern bearing are simulated, which provides experimental basis for the dynamic research of water-lubricated bearing. Collecting multi-sectional journal displacement data at different rotational speeds and establishing a bearing film thickness identification model, and limited measurement points can identify the dynamic film thickness data in the circumferential direction of bearing section. The three-dimensional film thickness distribution of the full bearing is obtained by combining the FE simulation and PSO algorithm with the proposed film thickness identification model as a way to study the influence of different rotational speeds on the distributed lubrication characteristics of WSB under offset load.

Results show that the maximum identification error of the bearing film thickness identification model is less than 7%; the test results are in good agreement with the identification results. The various parts of the tail bearing show different patterns of film thickness distribution under offset load. With the increase of rotational speed, the minimum film thickness of D_2_ section increases significantly more than other sections, and the maximum value reaches 27.82 µm. The mid-arch phenomenon of the journal is aggravated, and the hydrodynamic lubrication effect in the middle of the bearing is enhanced. The lubrication state presents dynamic zoning characteristics for WSB under offset load, large length-to-diameter ratio, and soft materials. Each lubrication sub-region area non-linear changes, when the speed increases from 35 rpm to 175 rpm, the hydrodynamic lubrication sub-region expands by 12.6%, and the boundary lubrication and mixed lubrication sub-regions decrease by 11.9% and 33.8%, respectively, indicating that the rotational speed has a significant effect on the distributed lubrication characteristics of WSB. Research results are instructive for analyzing the bearing lubrication status and, thus, predicting the service life of the shaft system.

However, the film thickness identification method still needs further optimization. The presence of rough contact in the main wear area near the ballast end affects the identification results of film thickness, so future work will consider the introduction of more measurement points and in-depth consideration of the effects of bearing wear and deformation.

## Figures and Tables

**Table 1:** Main parameters of the test bearing.

Parameters	Symbols and Units	Value
Lining diameter	*d*_1_*/*mm	324
Sleeve diameter	*d*_2_*/*mm	403.68
Lining thickness	*l*_1_/mm	20
Sleeve thickness	*l*_2_*/*mm	39.84
Bearing length	*L*/mm	1200
Radius clearance	*c/*mm	0.5
Length diameter ratio	—	3.7
Groove radius	*r/*mm	94.5
Groove position	*e/*mm	79.5

**Table 2:** Sensor parameters.

Sensor Model	2200-U05	3010-U3
Measurement range	0.5 mm	3 mm
Zero Point	0.05 mm	0.3 mm
Absolute error	≤±1 μm	≤±7.5 μm
Resolution	0.025 μm	0.15 μm
Temperature stability	≤±0.075 μm	≤±0.75 μm
Probe shell material	Stainless steel and ceramics	Stainless steel and plastics

**Table 3:** Test-condition parameter.

Test Condition	Value
Load	75 kN
Water supply temperature	25 °C
Water supply flow	41 L/min
Water supply pressure	0.13 MPa
Rotational speed	15~215 rpm

**Table 4:** Lubrication sub-region range at each speed.

Rotating Speed	Boundary Lubrication Sub-Region Range	Mixed Lubrication Sub-Region Range	Hydrodynamic Lubrication Sub-Region Range
35 rpm	375 mm	120 mm	705 mm
65 rpm	310 mm	133 mm	757 mm
95 rpm	221 mm	121 mm	858 mm
135 rpm	196 mm	107 mm	897 mm
175 rpm	164 mm	98 mm	938 mm

## Data Availability

The data presented in this study are available on request from the corresponding author. The data are not publicly available due to privacy.
